# Healthy-related quality of life in patients with cervical cancer in Southwest China: a cross-sectional study

**DOI:** 10.1186/s12913-021-06723-7

**Published:** 2021-08-19

**Authors:** Min Zhao, Lei Luo, Chun-hong zhang, Jin-ping zhang, Jia-yan Yuan, Rong-yan Gu, Song-rui Ding

**Affiliations:** 1 Medical Administration Department, The Third Affiliated Hospital of Kunming Medical University (Yunnan Cancer Hospital), 519Kun Zhou Road, Xi Shan county, Kunming, 650118 Yunnan China; 2 School of Public Health, Kunming Medical University, 1168 Yu Hua Street Chun Rong Road, Cheng Gong New City, Kunming, 650500 Yunnan China

**Keywords:** Cervical cancer, Health-related quality of life, Influencing factors, FACT-cx (4.0)

## Abstract

**Background:**

Cervical cancer is the second most common female malignant tumor in the world. According to a study in 2018, the incidence of cervical cancer in Yunnan Province of China was 11.42 per 100,000, the mortality rate was 3.77 per 100,000, and higher than the national average. Health-related quality of life (HRQoL) can be used not only in the selection and effect evaluation of clinical treatment plans of cervical cancer, but also in the evaluation of prognosis and long-term survival status. In this study, 288 cervical cancer patients admitted to the Yunnan Cancer Hospital in Southwest China from 2018 to 2020 were used as the survey objects to understand the HRQoL of cervical cancer patients and explore the related factors that affect HRQoL.

**Methods:**

The Chinese version of the functional assessment of cancer therapy-cervix (functional assessment of cancer therapy-cervix v4.0, FACT-Cx V4) was used to investigate 288 patients with cervical cancer in Yunnan Province. Statistical analysis was performed using t-test, analysis of variance, multiple linear regression and other methods.

**Results:**

The total FACT-Cx score of cervical cancer patients was (130.16 ± 14.20), the physical well-being (PWB) score was (22.02 ± 4.47), the social/family well-being (SWB) score was (25.66 ± 3.59), the emotional well-being (EWB) score was (19.75 ± 3.54), the functional well-being (FWB) score was (16.91 ± 5.01) and the additional focus area (cervical cancer subscale, CxS) score was (45.78 ± 4.61). From the multi-factor analysis results, the scores of PWB, FWB, Cxs and the total FACT-Cx were related to the choice of different treatment methods, the PWB scores of patients with concurrent chemoradiotherapy was low(β = − 1.67, *P* = 0.003), the FWB scores of patients with concurrent chemoradiotherapy was low(β = − 2.02, *P* = 0.001), the CxS scores of patients with concurrent chemoradiotherapy was low(β = − 1.61, *P* = 0.006), the total score of FACT-Cx of patients with concurrent chemoradiotherapy was low(β = − 5.91, *P* = 0.001). SWB score was affected by marital status, married patients had high PWB scores(β = 5.44, *P* = 0.006). The patients with heavy disease expenditures as aproportion of family disposable income(β = − 3.82, *P* = 0.002) and aged 60 and above(β = − 3.29, *P* = 0.003) had lower FWB scores. The total score FACT-Cx of patients participating in cervical cancer screening was higher(β = 7.61, *P* = 0.001).

**Conclusion:**

The choice of treatment method is the common influencing factor of PWB, FWB, Cxs and the total FACT-Cx. Disease expenditures as a proportion of family disposable income, the treatment method, the marital status and whether to participate in cervical cancer screening affect the patient’s evaluation of their own HRQoL. Medical staff should pay special attention to the choice of different treatment methods, popularize vaccination knowledge and cervical cancer screening, give more humanistic care and health education to cervical cancer patients who have low education level, poor economic conditions, divorced or separated, and encourage patients to participate in active treatment to improve the health-related quality of life.

## Background

Cervical cancer is the second most common female malignant tumor in the world [1], which is a serious threat to women’s health and is also one of the most important public health problems among adult women in the world [2]. In 2018, there were an estimated 570,000 cases and 311,000 deaths of cervical cancer worldwide, with an incidence rate of 15.3 per 100,000 and a mortality rate of 7 per 100,000 [3]. In China, according to a 2018 study, the number of new cervical cancer cases was 106,400 [4], the incidence rate was 10.88 per 100,000, and the mortality rate was 3.17 per 100,000, accounting for the 8th place in female cancer mortality [5], which showed a gradual downward trend [6]. Yunnan Province located on the Yunnan-Guizhou Plateau in southwestern China, that has an altitude of more than 2000 m and is the region with the largest concentration of ethnic minorities in China [7]. In 2018, the incidence of cervical cancer in Yunnan Province was 11.42 per 100,000 and the mortality rate was 3.77 per 100,000, and higher than the national average [8], the average age of onset of cervical cancer in Yunnan Province has gradually shown an upward trend in the past 5 years [9].

With the transformation of the biological-psychological-social medical model, the treatment goal of cervical cancer has also been elevated to the improvement of the quality of life [10]. Therefore, in addition to controlling tumors and improving the survival rate of patients, maintaining and improving the health-related quality of life (HRQoL) of cervical cancer patients has become an important issue for medical service providers [11]. HRQoL can be used not only in the selection and effect evaluation of clinical treatment plans, but also in the evaluation of prognosis and long-term survival status [12]. More and more people are also paying attention to the HRQoL of patients who survive cervical cancer [13]. For example, fregnani C [14] used FACT-Cx scale to evaluate the HRQoL of patients with cervical cancer in Brazil, Larissa et al. [15] studied the quality of life and related factors of patients with cervical cancer. However, there are few studies on the HRQoL of patients with cervical cancer in Southwest China, the HRQoL of patients with cervical cancer is related to many factors. It is very important to pay attention to improve the health-related quality of life of cervical cancer patients [16]. Therefore, this study evaluated the health quality of life of patients with cervical cancer in Southwest China by FACT-Cx scale, and explored its influencing factors, so as to provide scientific basis for prolonging the survival time and improving health quality of life of patients with cervical cancer in Southwest China.

## Objects and methods

### Research subjects

This study was a cross-sectional study. All cervical cancer patients (288 cases) who were admitted and clearly diagnosed at the Third Affiliated Hospital of Kunming Medical University (Yunnan Cancer Hospital) from January 2018 to December 2020 were included in the survey. The survey was completed and the questionnaire response rate was 100%.

Target inclusion criteria: (1) Cervical cancer patients diagnosed pathologically, able to understand the questions raised by the investigator; (2) Cervical cancer patients are informed consent and cooperate with the investigation; (3) Those who had no previous or current mental illness or impaired consciousness.

Target exclusion criteria: (1) Cervical cancer patients with cognitive impairment, unconsciousness and inability to express their feelings clearly; (2) Critical patients; (3) Company with other malignant tumors or serious illness.

The investigators in this study were all medical students with a medical background or medical personnel, and they have undergone uniform training before the investigation. They were aware of the meaning and filling standards of each item in the scale.

### The research method

Collecting the basic characteristics of patients through questionnaires (age, education level, occupation, marital status, medical insurance type, number of existing children, family income, disease expenditures as a proportion of family disposable income, menopausal status, contraceptive methods, whether they were aware of the cervix Cancer screening, whether to participate in cervical cancer screening, whether to know about HPV vaccine, and how to find the disease); checking the case data to collect the clinical diagnosis and treatment characteristics of the patient from medical records (clinical stage, treatment method), and adopting the cervical cancer scale (functional assessment of cancer therapy-cervix v4.0, FACT- Cx V4) developed by the Center for Outcome Research and Education in the United States. In the evaluation of the Chinese version of the quality of life scale for cancer patients, Wan Chonghua et al. [17] confirmed that the scale has good reliability, validity, responsiveness and feasibility, and can be used as an evaluation tool for the quality of life of cancer patients in China. In this study, we conducted a pre survey on 68 patients in October 2017, which also confirmed that the scale has good reliability and validity. As a doctor, the investigator explained the purpose, content and requirements of the survey to the patient. After obtaining the patient’s informed consent, the investigator will investigate the patient and fill in the scale. The investigator will check the completeness of the scale when the scale was returned. .

### Scale scoring method FACT-cx (V4.0)

Chinese version scale includes general module (general module of the functional assessment cancer therapy, FACT-G) and additional attention module for cervical cancer (cervical cancer subscale, CxS), a total of 5 dimensions, 42 items. FACT-G includes 4 dimensions and 27 items: 7 items for physical well-being (PWB), 7 items for social/family well-being (SWB), and emotional well-being (emotional well-being). EWB) 6 items, functional well-being (functional well-being, FWB) 7 items. There are 15 items in CxS, including symptoms, side effects and psychological activities, etc. (The abbreviations are PWB, SWB, EWB, FWB, CxS).

#### Calculation of item score

The items of the scale are answered as follows: not at all, a little, some, equivalent, very. About positive items, the higher the level, the higher the health-related quality of life. There are 20 positive items in the scale, the scoring rules for each item are 0 points, 1 point, 2 points, 3 points, 4 points. In addition, there are 22 reverse items, and the scoring rules for each item are 4 points, 3 point, 2 points, 1 points, 0 points..

#### Dimensions and Total scale score

The FACT scale score ranges from 0 to 168. The dimension score is calculated by adding the scores of all items in each dimension. The sum of the scores of each dimension is the total score of the scale. The higher the score, the better the health-related quality of life of the research object.

### Statistical analysis

Adopting Epidata 3.1(Epidata Entry 3.1.2701.2008) to input valid questionnaire information, all of the analyses were performed using IBM SPSS Statistics for Windows, Version 23.0 (IBM Corp, Armonk, NY, USA). The total score of the scale was normally distributed. In the single factor analysis, the comparison of the two sets of sample means used t-test, and the comparison of multiple sets of sample means used one-way variance analyze, then using LSD-t test to make pairwise comparisons. Using multiple linear regression analysis method, the dependent variable was the score of each dimension of the scale and the total score of the scale, the independent variable was the influencing factor in the single factor analysis, and the method of independent variable entering the model was stepwise regression, and the inclusion criterion α was ≤0.05, the exclusion criterion α was ≥0.10. Inspection level α = 0.05.

## Results

### General situation

In this study, a total of 288 research subjects, of which 39.9% received radiotherapy and chemotherapy. At the time of the interview, 26.7% of these women had not completed primary education, 71.2% were farmers, 89.9% were married, 74.0% were not aware of cervical cancer screening, and 94.1% believed that the disease expenditures as a proportion of family disposable income were medium and heavy. The main way to discover disease was through clinical symptoms(84.4%). The most commonly used treatment methods were radical hysterectomy(45.8%) and Concurrent chemoradiotherapy(39.9%).See Table 1.

**Table 1:** Social demographics and clinical characteristics of the research population

Variables	n	%
**Age group (years)**		
≤ 39	38	13.2
40–59	206	71.5
≥ 60	44	15.3
**Level of education**		
Below Primary school	77	26.7
Primary school	100	34.7
Junior middle school	80	27.8
High school and above	31	10.8
**Profession**		
Business unit	11	3.8
Enterprise staff	26	9.0
Freelancer	13	4.5
Farmer	205	71.2
Unemployed	20	6.9
Retirement	13	4.5
**Marital status**		
Unmarried	3	1.0
Married	259	89.9
Widowed	17	5.9
Divorced	9	3.1
**Health care type**		
Non-Employee medical insurance	249	86.5
Employee medical insurance	39	13.5
**Number of children**		
None	5	1.7
1–2	208	72.2
≥ 3	75	26.0
**Household income($)**		
0–391	108	37.5
392–783	89	30.9
784–1174	45	15.6
1175–1566	13	4.5
≥ 1567	33	11.5
**Disease expenditures as a proportion of family disposable income**		
little	17	5.9
Medium	74	25.7
Heavy	197	68.4
**Menopause**		
Yes	145	50.3
No	143	49.7
**contraception**		
IUD	184	63.9
Condoms	25	8.7
Oral contraceptive	8	2.8
Ligation	37	12.8
Other	2	0.7
None	32	11.1
**Are you know of cervical cancer screening**		
Yes	75	26.0
No	213	74.0
**Whether to participate in cervical cancer screening**		
Yes	45	15.6
No	243	84.4
**Whether to know about the HPV vaccine**		
Yes	78	27.1
No	210	72.9
**Ways to discover disease**		
Clinical symptoms	243	84.4
Physical examination	20	6.9
Screening	25	8.7
**Clinical stages**		
Phases IA	10	3.5
Phases IB	131	45.5
Phases IIA	44	15.3
Phases IIB	44	15.3
Phases III	47	16.3
Phases IV	12	4.2
**Treatment**		
Radical hysterectomy	135	46.9
Concurrent chemoradiotherapy	115	39.9
Adjuvant treatment after surgery	38	13.2

The Table 2 indicate scores of the subjects in each dimension and the scores of the FACT-Cx (4.0) scale. The total FACT-Cx score of women with cervical cancer was (130.16 ± 14.20), the PWB score was (22.02 ± 4.47), and the SWB score was (25.66 ± 3.59)) score, EWB score was (19.75 ± 3.54), FWB score was (16.91 ± 5.01), CxS score was (45.78 ± 4.61).

**Table 2:** Health-related quality of life scores for cervical cancer patients

FACT-Cx	Mean (SD)	Median	Minimum	Maximum	Score range
Physical well-being (PWB)	22.02(4.47)	23.0	8.0	28.0	0–28
Social/Family well-being (SWB)	25.66(3.59)	27.0	11.0	28.0	0–28
Emotional well-being (EWB)	19.75(3.54)	20.0	7.0	24.0	0–24
Functional well-being (FWB)	16.91(5.01)	17.0	2.0	28.0	0–28
Cervical cancer subscale (CxS)	45.78(4.61)	47.0	28.0	53.0	0–60
Total FACT-Cx	130.16(14.20)	132.0	83.0	157.0	0–168

### Univariate analysis of patient quality of life

The differences in total scores of EWB score and FACT-Cx by age group were all statistically significant (*P* < 0.05). Women under 39 years of age had the best HRQoL. The difference of educational level in the total score of FWB and fact CX was statistically significant (*P*<0.05), and women with a high school education level or above had the highest HRQoL. The difference in marital status between SWB and FACT-Cx total scores was statistically significant (*P*<0.05). HRQoL of married patients was higher than other marital status. Disease expenditures as a proportion of family disposable income was related to the FWB score of patients (*P*<0.05). Menopausal status was related to total score of SWB, FWB and FACT-Cx of patients (*P*<0.05). Knowledge of cervical cancer screening and participation in cervical cancer screening were both related to the scores of some dimensions except SWB and EWB(*P*<0.05), the scores of those who participated in the screening were higher than those who did not participate in the screening. The treatment method was related to the scores of some dimensions except EWB. (*P* < 0.05). See Table 3.

**Table 3:** Association of health-related quality of life, social demography, and clinical variables (univariate analysis)

	Physical well-being (PWB)			Social/Family well-being (SWB)			Emotional well-being (EWB)			Functional well-being (FWB)			cervical cancer subscale (CxS)			Total FACT-Cx		
	Mean (SD)	t/F	***p***-value	Mean (SD)	t/F	***p***-value	Mean (SD)	t/F	***p***-value	Mean (SD)	t/F	***p***-value	Mean (SD)	t/F	***p***-value	Mean (SD)	t/F	***p***-value
**Age group (years)**																		
≤ 39	23.34(3.69)	2.99	0.052	26.61(2.97)	1.52	0.221	20.79(3.01)	4.20	0.016*	19.79(4.81)	7.56	0.001**	45.92(3.88)	0.44	0.647	136.45(12.63)	5.24	0.006**
40–59	21.63(4.64)			25.52(3.67)			19.37(3.67)			16.47(5.08)			45.64(4.80)			128.68(14.64)		
≥ 60	22.70(4.06)			25.50(3.60)			20.61(3.02)			16.50(3.98)			46.34(4.35)			131.66(11.83)		
**Level of education**																		
Below Primary school	21.88(4.53)	1.81	0.146	25.04(3.91)	1.19	0.315	19.91(3.36)	2.33	0.074	15.49(4.31)	10.36	< 0.001**	46.26(4.74)	1.36	0.257	128.64(13.07)	4.89	0.002**
Primary school	21.33(4.75)			25.74(3.72)			19.09(3.59)			15.82(4.48)			45.04(4.94)			127.02(15.28)		
Junior middle school	22.68(4.23)			26.06(3.00)			20.46(2.99)			18.76(4.94)			46.16(4.20)			134.18(11.20)		
High school and above	22.90(3.79)			25.94(3.67)			19.65(4.71)			19.19(6.20)			46.03(4.09)			133.71(17.37)		
**Profession**																		
Business unit	23.64(4.80)	0.92	0.471	24.55(5.77)	0.34	0.886	18.00(5.22)	1.53	0.18	18.00(4.82)	2.96	0.013*	44.73(5.18)	0.90	0.482	128.91(21.82)	0.50	0.773
Enterprise staff	22.81(3.23)			26.04(2.95)			20.81(3.31)			19.69(4.69)			44.38(4.91)			133.73(13.19)		
Freelancer	21.54(4.75)			25.69(3.82)			19.54(4.01)			18.62(4.37)			45.46(3.95)			130.85(14.01)		
Farmer	22.05(4.57)			25.71(3.49)			19.87(3.27)			16.28(5.00)			46.11(4.67)			130.06(13.94)		
Unemployed	20.75(5.30)			25.20(4.35)			18.80(3.65)			17.25(3.52)			45.55(3.10)			127.55(13.41)		
Retirement	21.08(2.75)			25.77(2.95)			18.85(5.23)			18.23(6.34)			45.00(5.12)			128.92(15.39)		
**Marital status**																		
Unmarried	24.33(3.21)			20.67(7.02)			22.33(2.08)			19.00(2.65)			46.00(3.61)			132.33(16.29)		
Married	22.08(4.51)			26.10(3.29)			19.75(3.49)			16.97(4.85)			45.9(4.48)			130.83(13.95)		
Widowed	21.41(3.92)			21.76(3.54)			19.88(3.41)			16.71(5.77)			45.76(4.47)			125.53(13.52)		
Divorced	20.78(4.84)	0.61	0.606	22.00(3.32)	14.99	< 0.001**	18.67(5.24)	0.82	0.484	15.11(8.15)	0.58	0.629	42.33(7.65)	1.75	0.156	118.89(17.92)	2.76	0.043*
**Health care type**																		
Non-Employee medical insurance	21.93(4.62)	0.80	0.373	25.65(3.55)	0.01	0.919	19.80(3.35)	0.30	0.585	16.57(4.86)	9.08	0.003**	45.98(4.48)	3.46	0.064	129.96(13.81)	0.36	0.547
Employee medical insurance	22.62(3.41)			25.72(3.87)			19.46(4.60)			19.13(5.41)			44.51(5.26)			131.44(16.67)		
**Number of children**																		
None	23.80(1.64)	0.40	0.669	21.60(5.55)	3.82	0.023*	22.00(1.58)	1.03	0.359	18.80(1.64)	2.26	0.107	43.00(6.67)	1.09	0.337	129.20(11.71)	0.64	0.529
1–2	22.00(4.45)			25.86(3.31)			19.71(3.63)			17.23(5.22)			45.93(4.40)			130.75(14.22)		
≥ 3	21.96(4.67)			25.39(4.04)			19.71(3.35)			15.92(4.40)			45.57(5.03)			128.60(14.36)		
**Household income($)**																		
0–391	21.67(4.50)	1.31	0.266	25.65(3.57)	0.26	0.902	19.47(3.35)	0.57	0.686	16.24(4.63)	1.79	0.130	46.22(4.35)	3.24	0.013*	129.29(12.81)	1.73	0.143
392–783	22.08(4.49)			25.56(3.74)			19.83(3.50)			16.73(5.10)			45.82(4.95)			130.07(14.98)		
784–1174	21.93(4.44)			25.42(3.89)			19.67(4.13)			17.27(5.06)			44.44(5.08)			128.73(16.04)		
1175–1566	21.00(5.40)			26.15(2.76)			19.85(3.74)			18.00(3.56)			43.00(4.53)			128.00(14.12)		
≥ 1567	23.55(3.87)			26.12(3.19)			20.52(3.40)			18.70(5.99)			47.18(2.94)			136.06(13.10)		
**disease expenditures as a proportion of family disposable income**																		
little	22.76(4.52)	0.99	0.373	26.06(2.33)	0.11	0.895	19.59(4.02)	0.63	0.533	19.88(5.33)	10.06	< 0.001**	45.12(4.73)	0.38	0.686	133.41(13.46)	2.80	0.062
Medium	22.51(4.49)			25.66(3.69)			20.15(3.39)			18.49(4.78)			46.11(4.51)			132.97(13.32)		
Heavy	21.77(4.46)			25.63(3.65)			19.61(3.56)			16.07(4.84)			45.72(4.65)			128.82(14.45)		
**Menopause**																		
Yes	21.74(3.94)	1.17	0.281	25.21(3.51)	4.65	0.032*	19.77(3.56)	0.01	0.940	16.01(4.76)	9.87	0.002**	45.66(4.85)	0.21	0.650	128.41(13.25)	4.47	0.035*
No	22.31(4.96)			26.12(3.62)			19.73(3.53)			17.83(5.09)			45.91(4.37)			131.93(14.95)		
**contraception**																		
IUD	21.80(4.60)	0.57	0.721	25.71(3.57)	0.16	0.978	19.59(3.70)	0.92	0.469	16.50(4.85)	4.00	0.002**	45.72(4.84)	0.44	0.823	129.34(14.42)	1.39	0.228
Condoms	23.28(3.61)			25.68(4.17)			20.28(3.70)			20.56(5.38)			46.04(4.97)			135.84(15.36)		
Oral contraceptive	22.25(4.13)			25.63(2.33)			18.25(1.49)			16.13(4.61)			45.88(2.70)			128.13(11.15)		
Ligation	21.86(4.80)			25.38(3.74)			19.70(3.37)			15.73(4.66)			45.54(4.34)			128.22(14.23)		
Other	23.50(2.12)			27.50(0.71)			21.00(0.00)			21.00(2.83)			42.00(1.41)			135.00(1.41)		
None	22.34(4.21)			25.63(3.52)			20.63(3.01)			17.75(5.02)			46.44(3.76)			132.91(12.23)		
**Are you know of cervical cancer screening**																		
Yes	23.09(4.00)			26.01(3.23)			20.12(3.81)			17.91(5.07)			46.99(3.95)			134.12(13.59)		
No	21.64(4.58)	5.93	0.016*	25.54(3.70)	0.97	0.326	19.62(3.44)	1.11	0.293	16.56(4.95)	4.04	0.045*	45.36(4.76)	7.03	0.008**	128.77(14.18)	8.08	0.005**
**Whether to participate in cervical cancer screening**																		
Yes	23.73(3.90)			26.36(3.03)			20.67(3.32)			18.62(4.54)			47.20(4.28)			136.58(12.86)		
No	21.70(4.51)	8.00	0.005**	25.53(3.67)	2.00	0.159	19.58(3.56)	3.61	0.058	16.60(5.03)	6.33	0.012*	45.52(4.63)	5.10	0.025*	128.97(14.15)	11.28	0.001**
**Whether to know about the HPV vaccine**																		
Yes	21.74(4.11)			25.74(3.48)			19.81(3.80)			18.03(5.51)			46.05(4.42)			131.37(14.11)		
No	22.12(4.61)	0.41	0.523	25.63(3.63)	0.05	0.817	19.73(3.45)	0.03	0.866	16.50(4.75)	5.36	0.021*	45.69(4.69)	0.36	0.551	129.71(14.25)	0.78	0.378
**Ways to discover disease**																		
Clinical symptoms	22.02(4.40)	0.99	0.372	25.63(3.61)	0.05	0.948	19.75(3.44)	0.53	0.592	16.82(4.97)	2.56	0.079	45.47(4.61)	3.81	0.023*	129.72(13.81)	2.03	0.133
Physical examination	20.95(5.03)			25.85(3.20)			19.15(4.34)			15.65(5.35)			47.00(3.67)			128.80(15.37)		
Screening	22.84(4.71)			25.80(3.80)			20.24(3.87)			18.84(4.76)			47.84(4.80)			135.56(16.37)		
**Clinical stages**																		
Phases IA	25.40(3.57)	1.82	0.109	26.80(2.57)	0.98	0.429	22.50(2.84)	2.08	0.068	21.40(3.34)	2.12	0.063	49.00(4.29)	1.92	0.09	145.10(12.83)	2.46	0.033*
Phases IB	22.24(4.82)			25.41(3.96)			19.37(3.70)			16.95(5.28)			45.82(4.37)			129.85(14.56)		
Phases IIA	22.18(3.73)			25.73(2.94)			19.95(3.05)			16.68(4.29)			46.07(4.66)			130.61(12.90)		
Phases IIB	21.48(4.32)			25.20(4.18)			20.34(3.21)			15.84(5.25)			46.27(3.70)			129.14(13.42)		
Phases III	21.04(4.44)			26.15(2.90)			19.77(3.64)			17.19(3.96)			44.57(5.64)			128.72(14.03)		
Phases IV	22.08(3.42)			27.00(1.41)			18.58(3.87)			16.42(6.80)			44.67(4.91)			128.75(15.08)		
**Treatment**																		
Radical hysterectomy	22.05(5.08)			25.42(3.02)			18.84(3.82)			17.42(5.70)			45.68(4.30)			129.42(15.45)		
Concurrent chemoradiotherapy	23.12(4.16)			25.93(3.51)			20.21(3.35)			17.77(4.77)			46.84(4.13)			133.90(13.19)		
Adjuvant treatment after surgery	24.87(3.49)			26.76(2.22)			19.61(3.45)			19.97(4.78)			47.07(4.25)			138.29(11.97)		

*:*P* < 0.05, **:*P* < 0.01

### Multiple linear regression of factors affecting quality of life

PWB score was affected by treatment methods, the PWB scores of patients with concurrent chemoradiotherapy was low(β = −1.67, *P* = 0.003). SWB score was affected by marital status, married patients had high PWB scores(β = 5.44, *P* = 0.006). FWB score was affected by education level, disease expenditures as a proportion of family disposable income, treatment, whether to participate in cervical cancer screening and age group. The patients with junior middle school(β = 3.27, *P <* 0.001), high school and above(β = 3.70, *P <* 0.001) and cervical cancer screening(β = 2.03, *P* = 0.012) had higher FWB scores; The patients with heavy disease expenditures as aproportion of family disposable income(β = −3.82, *P* = 0.002), concurrent chemoradiotherapy(β = −2.02, *P* = 0.001), aged 40 to 50(β = −3.32, *P <* 0.001) and aged 60 and above(β = −3.29, *P* = 0.003) had lower FWB scores. The score of CxS was affected by the way of disease discovery and treatment methods, the patients who found the disease by screening had higher CxS scores(β = 2.37, *P* = 0.014), the CxS scores of patients with concurrent chemoradiotherapy was low(β = −1.61, *P* = 0.006). The total score of FACT-Cx was affected by treatment, whether to participate in cervical cancer screening, the total score of FACT-Cx of patients with concurrent chemoradiotherapy was low(β = −5.91, *P* = 0.001), the total score FACT-Cx of patients participating in cervical cancer screening was higher(β = 7.61, *P* = 0.001). See Table 4.

**Table 4:** Factors associated with health-related quality of life in women with cervical cancer

Scale dimensions	Domains of quality of life	Difference in means (βcoefficient)	***R***^**2**^	95%CI	***P***-value
**Physical well-being (PWB)**	**Treatment**				
	Concurrent chemoradiotherapy	−1.67	0.03	−2.78 - -0.57	0.003
	Postoperative adjuvant therapy	−0.73	0.03	−2.33-0.87	0.368
**Social/Family well-being (SWB)**	**Marital status**				
	Married	5.44	0.13	1.61–9.27	0.006
	Widowed	0.10	0.13	−3.03-5.23	0.601
	Divorced or separated	1.33	0.13	−3.06-5.73	0.551
**Functional well-being (FWB)**	**Level of education**				
	Primary school	0.33	0.10	−1.10-1.75	0.653
	Junior middle school	3.27	0.10	1.77–4.77	< 0.001
	High school and above	3.70	0.10	1.70–5.70	< 0.001
	**Disease expenditures as a proportion of family disposable income**				
	Medium	−1.40	0.07	−3.97-1.17	0.286
	Heavy	−3.82	0.07	−6.23 - -1.40	0.002
	**Treatment**				
	Concurrent chemoradiotherapy	−2.02	0.04	−3.25 - -0.78	0.001
	Postoperative adjuvant therapy	−0.34	0.04	−2.12-1.44	0.706
	**Whether to participate in cervical cancer screening**				
	Yes	2.03	0.02	0.44–3.61	0.012
	**Age group (years)**				
	40–50	−3.32	0.05	−5.02 - -1.62	< 0.001
	≧60	−3.29	0.05	−5.42 - -1.16	0.003
**cervical cancer subscale (CxS)**	**Discover disease patterns**				
	Physical examination	1.53	0.03	−0.56-3.62	0.152
	Screening	2.37	0.03	0.48–4.26	0.014
	**Treatment**				
	Concurrent chemoradiotherapy	−1.61	0.03	−2.75 - -0.47	0.006
	Postoperative adjuvant therapy	−0.86	0.03	−2.51-0.79	0.308
**Total FACT-Cx**	**Treatment**				
	Concurrent chemoradiotherapy	−5.91	0.04	−9.41 - -2.42	0.001
	Postoperative adjuvant therapy	−3.57	0.04	−8.63-1.48	0.165
	**Whether to participate in cervical cancer screening**				
	Yes	7.61	0.04	3.15–12.07	0.001

## Discussion

In most dimensions of HRQoL, except for women who underwent hysterectomy, women who are diagnosed earlier and treated for a longer period of time scored the highest [18]. Our results show that whether they are know of cervical cancer screening and whether they participate in cervical cancer screening affect the scores of some dimensions except SWB and EWB. The scores of patients who know and participate in cervical cancer screening are higher than those who do not know and do not participate in screening, and their HRQoL is also higher. It may be that patients who knowing and participating in cervical cancer screening can get konwledge of prvention of cervical cancer, which can early scanning, diagnosis and treatment, further improve their health quality of life, and have higher HRQOL scores. The occurrence and development of cervical cancer is related to cervical lesions, and it takes several to ten years from precancerous lesions to invasive cancer. But, some early lesions can be reversed [19]. In developed countries, the incidence of cervical cancer has dropped significantly, largely due to the early diagnosis and treatment of cervical precancerous lesions [20]. Therefore, strengthening the census and preventive education, expanding the awareness and participation rate of screening, and early detection and active treatment of precancerous lesions are the keys to the prevention and treatment of cervical cancer and improving the HRQoL of patients with cervical lesions [21].

In univariate and multivariate analysis, marital status affects the SWB evaluation of patients. The HRQoL of married patients is higher than that of single patients. Patients with stable marriage can get more family support during treatment, and they will get more satisfaction in terms of emotional comfort and financial support. Cancer patients have a long treatment cycle and suffer both physically and mentally during their illness. With the progress of the disease, patients who are unfortunately married or have no partners are more likely to have psychological problems such as depression and loneliness, which will affect the HRQoL level [22]. In our study, disease expenditures as a proportion of family disposable income affected the FWB score of patients. The higher the disease expenditures as a proportion of family disposable income, the lower the HRQoL score. It may be that patients whose disease expenditures accounts for a large proportion of family disposable income face greater economic pressure, resulting in more anxiety, poor sleep, poor work, unable to face their own diseases, and dissatisfied with the quality of life.

In the study of health-related quality of life of women with cervical cancer, Santos LD et al [23] concluded that the total score of FACT-Cx was 112.15 (22.91); Fregnani C et al [14] also found that the total score of FACT-Cx was 110.40 (25.60) in a study on the quality of life of cervical cancer in Brazil. The total score of FACT-Cx in this study is 130.16 (14.20), which is higher than previous studies. This difference may be related to the differences of different subjects and time periods. Our research results also show that the best HRQoL dimension is SWB(25.66(3.59)), which is consistent with the research results of Zhou et al. [22] and Ding et al [24] The worst score is in FWB(16.91(5.01)). We consider that different treatment methods and age affect the FWB score of patients, the FWB score of patients with concurrent radiotherapy and chemotherapy is lower than that of patients with surgery and postoperative adjuvant treatment. According to our research, due to the lack of early cervical cancer screening, most patients were diagnosed in the advanced stage. Platinum-based concurrent radiotherapy and chemotherapy are the standard treatment options for locally advanced cervical cancer, but this type of combination therapy is most often associated with higher toxicity and more intense side effects [14]. The side effects of long-term radiotherapy and multi cycle chemotherapy bring great pain and psychological trauma to patients [25]. Moreover, during waiting for radiotherapy, fear and misunderstanding of radiotherapy will cause anxiety, which causing physical symptoms and emotional distress will affect the health-related quality of life of patients [26]. The health-related quality of life of younger patients is also better than that of older patients, which may be related to the better physical function of younger patients [27].

The treatment of cervical cancer is based on the FIGO staging of the disease, including surgery and/or (chemical) radiotherapy [28, 29]. Depending on the treatment, there may be different side effects, such as bladder, bowel and vaginal dysfunction, lymphedema and lymphatic cyst [30, 31]. These side effects of effective treatments, as well as the emotional and social impact of the disease, will alway affect the patient’s HRQoL even if the survival period is prolonged [32]. In a study in Brazil, women who underwent hysterectomy showed better HRQoL scores [18], which is consistent with the conclusion of our study. In our study, the HRQoL scores of patients with cervical cancer undergoing surgery (Leep/radical hysterectomy) are higher than those of patients with concurrent radiotherapy and chemotherapy. According to a study in the United States, the difference of HRQoL in patients with early cervical cancer may be related to treatment, the HRQoL of patients receiving pelvic radiotherapy is different from that of patients receiving radical hysterectomy, especially the sexual function [33], radical hysterectomy may represent a way to improve HRQoL and sexual function of patients with early cervical cancer [34]. In a systematic review, it was also shown that radiotherapy was also associated with poor HRQoL [28]. In a survey by Osann et al. [35], it is observed that HRQoL of patients receiving radiotherapy (whether or not receiving chemotherapy) is worse than that of patients who just receive surgery.

## Conclusion

There are many factors affecting the health-related quality of life of cervical cancer patients, among which the choice of treatment method is the common influencing factor of PWB, FWB, Cxs and the total FACT-Cx. Disease expenditures as a proportion of family disposable income, the treatment method, the marital status and whether to participate in cervical cancer screening affect the patient’s evaluation of their own HRQoL. Medical staff should pay special attention to the choice of different treatment methods, popularize vaccination knowledge and cervical cancer screening, give more humanistic care and health education to cervical cancer patients who have low education level, poor economic conditions, divorced or separated, and encourage patients to participate in active treatment to improve the health-related quality of life.

## Limitation and innovation

Limitation: This study is a cross-sectional study, the causal relationship between HRQoL and the influencing factors of cervical cancer is difficult to determine. The sample size of this study is small, and the promotion of the research conclusion needs further verification.

Innovation: This is the first study on the HRQoL of cervical cancer patients in Southwest China, which can provide reference and policy guidance for improving the health related quality of life of cervical cancer patients in this region.

## Data Availability

The scale used in this study is reliable and applicable after reliability and validity test. The datasets generated and/or analysed during the current study are not publicly available due because they are related to patients but are available from the corresponding author on request.
